# Moving towards ICD-11 and DSM-V: Concept and evolution of psychiatric classification

**DOI:** 10.4103/0019-5545.58302

**Published:** 2009

**Authors:** P. K. Dalal, T. Sivakumar

**Affiliations:** Department of Psychiatry, C. S. M. Medical University, Lucknow - 226 003, UP, India

**Keywords:** Classification, clinical significance, comorbidity, dimensional model, discrete disease entity, disease, disorder, DSM, harmful dysfunction, ICD, polythetic, psychiatry, reification, syndrome, validity

## Abstract

A classification is as good as its theory. As the etiology of psychiatric disorders is still not clearly known, we still define them categorically by their clinical syndrome. There are doubts if they are valid discrete disease entities and if dimensional models are better to study them. We have come a long way till ICD-10 and DSM-IV, but there are shortcomings. With advances in genetics and neurobiology in the future, classification of psychiatric disorders should improve further. The concept, evolution, current status and challenges facing psychiatric classification are discussed in this review.

## INTRODUCTION

Diseases have existed since times immemorial and have not changed much. It is rather our understanding of diseases which has changed over time with medical advances. The evolution of our knowledge is reflected in our classificatory systems as well. Perhaps in no other branch of medicine is this as dramatically illustrated as in psychiatry. A study of concept and evolution of psychiatry classification reveals the progress made and predicaments ahead in our quest to understand disorders of the human psyche.

## CONCEPT OF DISEASE, SYNDROME AND DISORDER

Before studying about the intricacies of classification, it is essential to understand what is being classified in the first place.

Syndrome is a condition characterized by a particular symptom profile whose etiology, clinical significance or severity is variable.[1]

DISEASE is a condition with specific aetiopathogenesis. It has biomedical connotations.[1]

The term DISORDER, first introduced as a generic name for the unit of classification in DSM-I in 1952,[2] is a term midway between a disease or illness and a syndrome, in terms of consistency, correlates and significance.[1]

The typical progression of knowledge begins with the identification of clinical manifestations, i.e. syndrome, and the deviance from the “norm”; understanding of the pathology and etiology comes much later. There is no fixed point or agreed threshold of description beyond which a syndrome can be said to be a “disease”. With our current knowledge, Alzheimer's dementia is a condition that can be called a disease as its pathophysiology and tentative causes have been elucidated and proven. In contrast, schizophrenia still does not qualify as a disease.[2]

## CONCEPT OF CLASSIFICATION

Kendell described that every patient possesses three kinds of characteristics:

- Universal, shared with all others
- Shared with some but not others
- Unique, shared with no others

Where A dominates, classification is pointless; where C dominates, classification is impossible. The presence of B is the crucial one and so classification depends on B relative to A and C.[3]

Simply stated, Classification is the process by which the complexity of phenomena is reduced by arranging them into categories according to some established criteria for one or more purposes.[2]

Taxonomy is the metatheory of classification, including systematic study of the various strategies of classification.[2]

## PURPOSE OF MEDICAL/PSYCHIATRIC CLASSIFICATION

1. **Communication:** A classification enables users to communicate with each other about the disorders with which they deal. This involves using names of categories as standard shorthand ways of summarizing a great deal of information.[1] For example, when a patient is diagnosed as a case of moderate depressive episode (F32.1 in ICD-10), it implies that it is his first episode, there have been no manic or mixed episodes in the past, he is not psychotic and it is not substance induced, etc.
2. **Control:** Control of psychiatric disorders primarily refers to their treatment and prevention. This is the ultimate purpose of any classification.[1] In medicine, rheumatic fever, PANDAS and Sydenham's chorea are classified under postinfective autoimmune diseases. This means that they can be prevented by treating the infection properly. In psychiatry, psychotic disorders (be it a part of affective or nonaffective disorders or due to different etiologies) means antipsychotics need to be used. Similarly, a diagnosis of bipolar affective disorder requires usage of mood stabilizers.
3. **Comprehension:** Classification should provide comprehension or understanding of the causes of psychiatric disorders and the processes involved in their development and maintenance.[1] For example, in medicine, Diabetes mellitus is classified under endocrine disorders as a disorder of impaired glucose regulation on the basis of pathophysiology. Disorders can, of course, be treated without knowledge of their etiology or pathophysiology. Comprehension is not an end in itself but is desired in a classification because it usually leads to more effective control.[1]

ICD-10 and DSM-IV serve the purpose of communication well and to a certain extent control but not comprehension due to limitations in our understanding of psychiatric disorders.

## CLASSIFICATION IN PSYCHIATRY

Medical classification can be performed in several ways [Table 1].

**Table 1:** Comparison between medical and psychiatric classification

	Medical	Psychiatric
By clinical syndrome	Migraine	Schizophrenia
By pathological process	Ulcerative colitis	Alzheimer's dementia
By deviation from norm	Hypertension	Personality disorder
By hypothetical process	-	Dissociative disorder
By etiology	Tuberculosis	Posttraumatic stress disorder

Ideally, a classification of medical/psychiatric disorders should be based on knowledge of etiology or pathophysiology because this increases the likelihood of improving treatment and prevention efforts.[1]

Psychiatric disorders are unique in contemporary medicine because most of them are still defined and classified on the basis of their clinical syndromes. The human brain is an infinitely more complex machine with a much wider range of functions than the heart, the kidney or the liver. We therefore have more difficulty understanding its disorders. It may be a long time before we understand some of its more sophisticated functions, such as storing memories, emotions, decision making and generating and interpreting speech, and an even longer time before we understand the disorders of these remarkable abilities.[3]

The different taxonomic philosophies and strategies are[2]

1. **Classical taxonomic strategy:** Monothetic approach – The candidate must meet exactly the set of necessary and sufficient criteria that define a given class (e.g., periodic table of elements).
2. **Numerical taxonomy:** Polythetic approach – The candidate must meet a certain number of criteria to qualify for a diagnosis (e.g., diagnosis of schizophrenia in ICD-10 and DSM-IV).
3. **Prototype matching procedure:** Polythetic approach – Diagnosis is made according to how close a candidate meets a prototype.

Because of the intrinsically heterogeneous nature of psychopathology, psychiatric classifications are polythetic in nature.[2]

## WHAT CONSTITUTES A GOOD CLASSIFICATION OF PSYCHIATRIC DISORDERS?

1. **Reliability**[2]: It shows as to how far errors of measurement have been excluded from assessment. Diagnostic reliability can be improved by operational diagnostic criteria and by using structured interviews. Reliability establishes a ceiling for validity. Lower reliability means lower validity, but the converse does not hold true.
2. **Validity**[2]: How far a test actually measures what it is supposed to measure, meaning “the nature of reality”. It is an “all or none” concept.
3. **Utility**[2]: It is a graded concept and partially context dependent. The clinical utility of a classificatory system can be assessed empirically by taking into account its impact on three domains: Use, decision making process and clinical outcome.[4]
4. Ease of use.[2]
5. Applicability across settings and cultures.[2]
6. Meet needs of various users[2]: Clinicians, researchers and users of mental health services.

## PROBLEMS UNIQUE TO CLASSIFYING PSYCHIATRIC DISORDERS

- Psychiatry, in contrast to other branches of medicine, relies on the patient's own subjective report of symptoms and the doctor's observation of patient behavior to arrive at a diagnosis.[5]
- Psychiatry lacks objective and independent criteria for sorting out psychiatric disorders.[6]
- Psychiatric disorders are manifested by a quantitative deviation in behavior, ideation and emotion from a normative concept[1] and it is difficult to define normal human behavior.
- Psychiatric symptoms are highly nonspecific and quite unstable over time.[7]

## IMPORTANCE OF CLEARLY DEFINING A PSYCHIATRIC DISORDER

1. It influences estimates of psychiatric disorders in the community and allocation of tax-payer's money to manage them.[1]
2. It has potential legal implications in criminal cases[1] and in awarding disability benefits.
3. Lack of a clear conceptual definition can contribute to abuses of psychiatric diagnoses[8] as a means of controlling or stigmatizing socially undesirable behavior (e.g., misuse of psychiatric diagnosis in former USSR and China to jail dissidents of the system).
4. Lack of a proper definition of psychiatric disorder reduces confidence in our discipline among our colleagues and the general public.[1]

## EVOLUTION OF PSYCHIATRIC CLASSIFICATION

The first specific description of a mental illness appeared in approximately 3000 BC in a depiction of senile deterioration ascribed to Prince Ptah-hotep.[1] In India, in approximately 1400 BC, a classification of mental illness was included in Ayurveda.[1]

In the 1840 US census, number of individuals with mental illness or mental retardation were counted as a single category.[9] In the 1880 US census, seven distinct categories were recognized.[9] As there was no uniform accepted system of classification for a discipline that makes diagnosis on the basis of clinical signs and symptoms, each and every psychiatric hospital had its own way of diagnosis, which could even vary within the same hospital. By the turn of the 20^th^ century, there were almost as many statistical classifications in use as there were mental hospitals! This prevented accurate comparison among patient groups in different institutions.[9]

As the lack of a uniform classification ran the risk of discrediting psychiatry, the committee on statistics of the American medicopsychological association introduced a 22-item list of disorders in 1918.[9] In 1935, a revised and expanded version was incorporated into the second edition of the American Medical Association's standard classified nomenclature of diseases, which was suited for chronic inpatients.[9] As it was inadequate in other settings, by the end of World War II, there were four major competing classification systems in the US: The American Medical Association nomenclature, the US Army classification, the US Navy classification and a system developed for use in the veterans administration hospitals.[9] This again created the old problem and illustrated the need for a universally acceptable system of classification. The major landmarks in the subsequent evolution of psychiatric classification are illustrated in Flow chart 1.

**Flow chart 1:**
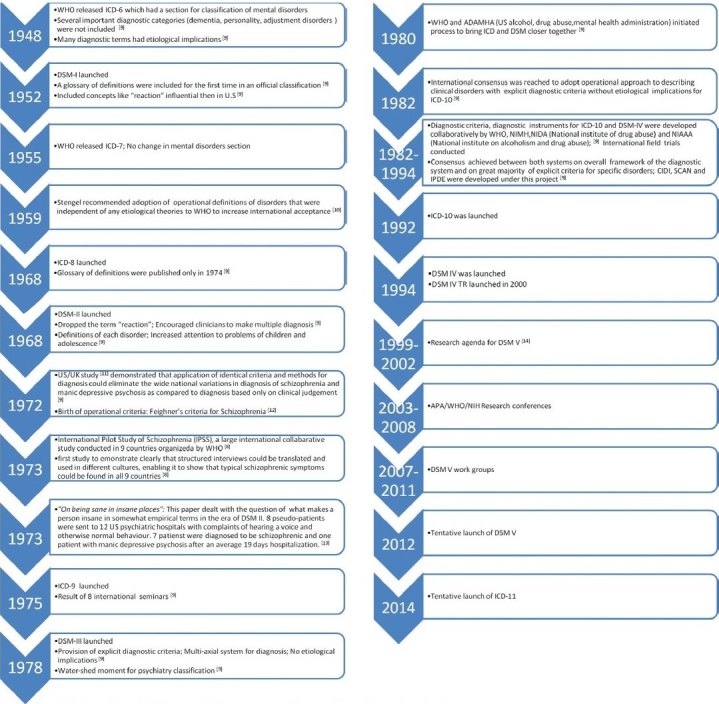
Evolution of ICD and DSM classification and important landmarks

Some salient differences between ICD-10 and DSM-IV are shown in Table 2.

**Table 2:** ICD-10 and DSM IV: Some salient differences

	ICD-10	DSM-IV
Origin[8]	International (WHO)	American Psychiatric Association
Comprehensiveness[2]	Comprehensive classification of all “diseases and related health problems”	Stand-alone classification of mental disorders
Presentation[8]	Different versions for clinical work research and use in primary care	A single document
Languages[8]	Available in all widely spoken languages	English version
Structure[8]	Part of overall ICD framework	
	Single axis in chapter V; separate multiaxial systems available	Multiaxial
Used in[2]	Most frequently used across the world for clinical work and training purposes	Designed, at least in the first instance, for use by American health professionals
Worldwide usage[2]	Every country is obliged to report basic morbidity data to WHO using its categories	Most frequently used in research work
Content[8]	Guidelines and criteria do not include social consequences of disorders	Diagnostic criteria usually include significant impairment in social functions

## WHAT HAVE WE ACHIEVED SO FAR?

When compared with the past, reliability of psychiatrist's diagnosis has dramatically improved. Current diagnostic categories are of great utility to practicing clinicians, epidemiology studies, clinical trials, etc., but not for researchers.

## WHY THE LONG INTERVAL FOR ICD-11 AND DSM-V?

Jablensky and Kendell list the following reasons for the long interval for ICD-11 and DSM-V:[2]

1. Frequent revisions may undermine assimilation by clinicians, hamper progress of research and damage credibility of our discipline.
2. Possibility of major research breakthroughs that may have a significant impact on nosology.
3. Satisfaction with performance of current systems.

## MEDICAL EVIDENCE -BASED MODEL FOR PSYCHIATRIC DISORDERS

Medical knowledge evolves from assigning a name to a group of phenomena and enriching the information content. There is an implicit assumption that the entities are discrete, with natural boundaries among them. With time, they are validated and research sheds light on their etiology and pathogenesis [Flow chart 2].

**Flow chart 2:**
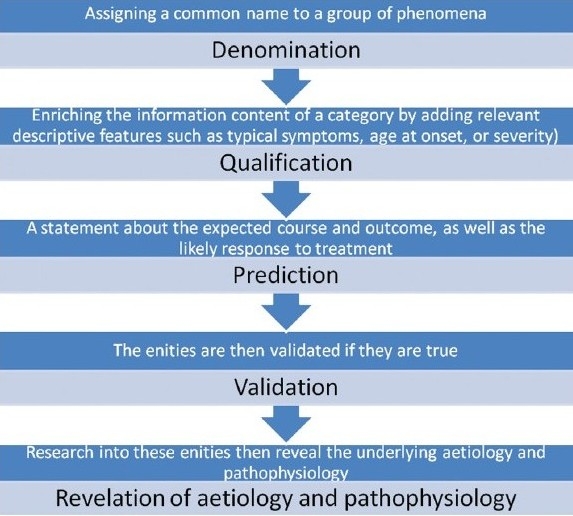
Evolution of knowledge about a disease entity

When categorical classification of psychiatric disorders was taken up, it was assumed that psychiatric disorders are also discrete entities and follow the same curve as medical diseases. Although discrete entity assumption has proved useful in identification of MECP2 gene for Rett's disorder,[15] it has not held true for several other psychiatric disorders.

## ARE PSYCHIATRIC DISORDERS REALLY DISCRETE ENTITIES?

Attempts to demonstrate natural boundaries between related syndromes (mania and schizophrenia) or a common syndrome and normality (depression, anxiety, personality) by locating a “zone of rarity” between them has failed. Several other DSM/ICD disorders have been found to cluster among the relatives of individuals with schizophrenia, major depression or bipolar affective disorder, and findings of such clusters have given rise to the concepts of “schizophrenia spectrum” and “affective spectrum” disorders.[16]

If disorders are independent categories, the coexistence of other disorders within a specific disorder should be just by chance. In fact, this is not the case. Psychiatric comorbidity seems to be the rule rather than the exception.[17] Across the lifespan, most individuals move in and out of comorbid diagnostic categories (e.g., anxiety disorders and depression). The separation between depression and anxiety has been criticized on many grounds. The most common form of affective disorder is actually mixed anxiety-depression,[18] comorbid anxiety and depression showed a greater stability in a 15-year follow-up study than each disorder alone.[19] Results from family and twin studies suggest common etiologic factors underlying both conditions.[19] Despite claims of specificity, the same classes of drugs have been increasingly used to treat the whole range of anxiety and depressive disorders.[19]

Although there is a growing assumption, at least within the research community, that most currently recognized psychiatric disorders are not disease entities, this belief has never been demonstrated, mainly because studies of the appropriate kind have rarely been mounted.[20]

Maj *et al.*, remarked that the lack of “zone of rarity” and high rates of comorbidities show that there are three possibilities:[21]

- Psychopathology does not consist of discrete disease entities as conceptualized by ICD-10 and DSM-IV.
- Psychopathology does consist of discrete disease entities, but these entities are not reflected by current diagnostic categories. If this is the case, current clinical research on “psychiatric comorbidity” may be helpful in the search for “true” disease entities, contributing in the long term to a rearrangement of present classifications, which may involve a simplification (i. e., a single disease entity may underlie the apparent “comorbidity” of several disorders), a further complication (i. e., different disease entities may correspond to different “comorbidity” patterns) or possibly a simplification in some areas of classification and a further complication in other areas.
- Nature of psychopathology is intrinsically heterogeneous, consisting in part of true disease entities and in part of reaction types or maladaptive response patterns. This is maladaptive response patterns. This is what Jaspers (1913) actually suggested when he distinguished between “true diseases” (such as general paresis), which have clear boundaries among themselves and with normality; “circles” (such as manicdepressive insanity and schizophrenia), which have clear boundaries with normality but not among themselves; and “types” (such as neuroses and abnormal personalities), which do not have clear boundaries either among themselves or with normality.

The current systems of ICD-10 and DSM-IV actively encourage multiple diagnoses in the same person, regardless of the possible contribution to etiology, allowing the maximum amount of diagnostic information. This has lead to an inflation of comorbidity rates (largely at the expense of artifactual comorbidity), a by-product of the splitting strategy adopted in ICD and DSM.[22] But, the importance given to personality disorders as comorbidity due to emergence of multiaxial classification systems is a positive development.

It is also well established that clinicians do not adequately capture the complexity of comorbidity, which may be due to the fact that they do not consider them to be a clinically relevant focus of treatment.[23] Desai opined that this was particularly relevant for making a diagnosis of personality disorders. He considered this important for developing countries where the human resource situation, and lack of appropriate data-capturing health information systems, may render the growing complexity of comorbidity impracticable, if not irrelevant.[23]

## VALIDITY OF ICD-10 AND DSM-IV DIAGNOSTIC CATEGORIES

The most important reason as to why a predominantly syndrome-based classification like that of psychiatry should be as valid as possible is that accurate delineation of clinical syndromes makes it easier to elucidate their etiology.[3] Robins and Guze validity criteria[24] for psychiatric disorders was based on a ‘discrete disease entity’ assumption. Our current knowledge indicates that there are shortcomings with their validity criteria [Table 3]. Several other authors have proposed their validity criteria for psychiatric disorders.

**Table 3:** Robins and Guze[24] criteria in the context of our current knowledge

Robins and Guze validity criteria (1970)[24]	Current scenario
Clinical description	Defined categorically by their clinical syndrome
Laboratory studies	No pathognomonic findings. Laboratory abnormalities are not specific to any psychiatric disorder
Delimitation from other disorders	High rates of nonspecific comorbidities.
	Comorbidity seems to be the rule rather than the exception[17]
Follow-up studies	Diagnostic instability for many disorders in epidemiologic studies. For example, a patient's diagnosis may change from recurrent brief depressive disorder to major depressive disorder to finally bipolar affective disorder or schizophrenia
Family studies	Wide variety of disorders may be expressed in the same family; eg., unipolar depressive disorder is the most common form of mood disorder found in families of bipolar probands[25]

1. Kendler's validity criteria (1980): Antecedent, concurrent and predictive validators.[26]
2. Andreason's additional validators (1995): Molecular genetics, molecular biology, neurochemistry, neuroanatomy, neurophysiology and cognitive neuroscience.[27]
3. Kendell and Jablensky (2003): One of two conditions be met
  - Zones of rarity between one syndrome and others
  - Clear qualitative differences in biological underpinnings of one syndrome and another.[16]

## PROBLEMS IN IDENTIFYING ETIOLOGY AND PATHOPHYSIOLOGY OF PSYCHIATRIC DISORDERS DEFINED BY ICD-10 AND DSM-IV

A particular profile of signs and symptoms can be the end result of different causes (e.g., depression can be unipolar, bipolar, drug induced, due to bereavement, post schizophrenic, etc.) and conversely a particular etiology can give rise to different profiles of signs and symptoms (e.g., same stressor can elicit different reactions in different persons or even in the same person at different times). Therefore, there is no one to one relationship between etiology and clinical presentation.

There is growing evidence that both genes and the brain circuits they regulate may underlie symptoms across many diagnostic categories.[6,28,29] The current classification system does not fully map on the neurobiology in terms of its pathophysiological groupings (e.g., OCD has a totally different neural circuit although it is grouped with anxiety disorders).[2] Despite beliefs of distinct mechanisms between schizophrenia and bipolar disorders, family studies have shown concurrent heritability.

The disorders of current classification are probably surface phenomena, resulting from multiple pathogenetic and pathoplastic interactions.[2] Two siblings with diagnosis of paranoid schizophrenia may have a completely different clinical picture as they may satisfy different criteria of the polythetic classification for the diagnosis to be made. This is a major reason for failure in major breakthroughs in genetics research, etc. as we compare dissimilar patients who have nothing in common except an ICD or DSM diagnostic category. This impedes our quest for etiology and pathophysiology of psychiatric disorders. This explains the search for simpler clues to genetic underpinnings, like the endophenotypes.[30]

## CATEGORICAL VS DIMENSIONAL MODELS

A fundamental choice in descriptive psychopathology classification is between a categorical and a dimensional structure. Although most sciences start with a categorical classification of their subject matter, they often replace this with dimensions as accurate measurement becomes possible.[31]

As discussed earlier, psychiatric disorders have traditionally been classified by dividing them into categories that are supposed to represent discrete clinical entities. All our knowledge about these disorders and clinical decision making to treat are based in this system. But, the categorical system is not without problems. Patients do not fit neatly into the available categories. Besides, there is the problem of comorbidities and doubts have been raised regarding the validity of these entities.

In the absence of clear evidence of points of rarity and discontinuities, the possibility of representing variation in symptomatology by dimensions rather than categories needs to be considered. Dimensional representation solves at a stroke all the problems associated with boundaries and comorbidity and may also be a powerful means of predicting outcome.[3]

The dimensional view of psychiatric disorders is comparable to that of hypertension and other medical diagnoses that are really extremes of normal distribution, and it reflects the nature of the underlying predisposition. Dimensional models characterize the subject by means of scores on two or more dimensions (e.g., Eysenck's three-dimensional personality model comprising pschoticism, neuroticism and introversion-extroversion).[8]

The problem with dimensions is that they are not of great value in clinical practice. For every patient, yes/no decisions need to be made, the most critical being whether the person requires treatment. This decision is, in turn, based on whether they have a disorder meriting treatment and, if so, which one. These clinical imperatives strongly favor categorical approaches to classification.[8]

There is no foreseeable prospect that any formal national or international classification will adopt a dimensional format, except possibly for personality disorders.[3] The rest of medicine is too firmly committed to categories, and in any case dimensions are better suited to the portrayal of variation in populations than day-to-day decisions about the treatment of individuals. This does not mean, however, that dimensional systems may not have an important role for research purposes or as an experimental alternative to an established typology. Indeed, they should be more widely used than they are.[3]

Taxometric results, reviewed by Haslam, favored categorical models for some disorders (like melancholia and eating disorders) and dimensional ones for others (like depression, generalized anxiety and posttraumatic stress disorder), supporting therefore a pluralistic view of psychiatric classification.[32]

## CURRENT DEFINITIONS OF PSYCHIATRIC DISORDERS: CLINICAL SIGNIFICANCE VS HARMFUL DYSFUNCTION

In ICD-10 and DSM-IV, the clinical significance criterion is used to establish the threshold for the diagnosis of a disorder in those situations in which the symptomatic presentation by itself (particularly in its milder forms) is not inherently pathological.[1]

This creates the problem of subthreshold states (in primary care, subthreshold disorders have been found to be associated with degrees of distress and disability comparable to full-fledged disorders. A recent WHO study emphasized that there are several-fold differences in the population prevalence of psychiatric disorders depending on whether “mild” cases are included or not[33]), difficulties in labelling normal reactions to stressful disorders[1] (bereavement, exam stress) and problems in diagnosing patients with clear-cut psychopathology but no clinical impairment[1] (e.g., a schizophrenia patient who gets commanding hallucinations from god but is functioning well without any distress or impairment).

In view of this problem, Wakefield has suggested that a concept of “harmful dysfunction” be used to define psychiatric disorders.[34] A “dysfunction” is construed as a failure of an internal mechanism to perform one of the functions for which it is naturally designed. For example, reading disorder is due to failure of an internal mechanism while illiteracy is not. “Harm” on the other hand, is understood as a value that is ascribed to that dysfunction depending on individual circumstances transforming the dysfunction into a disorder. This approach combines a factual scientific notion (dysfunction) with a value component (harm) to diagnose a disorder. Although the actual nature of the dysfunction may be unknown, Wakefield argues that it is possible for clinicians to judge whether symptoms are due to internal dysfunction. Unfortunately, Wakefield has yet to show that clinicians, for a variety of disorders, can distinguish normal reactions (e.g., depression after a loss) from harmful conditions that are the result of a dysfunction (e.g., the dysfunction of mood regulation in severe depression).[1]

## OTHER LIMITATIONS OF ICD-10 AND DSM-IV

### Developmental aspects not given due importance

ICD-10 and DSM-IV offer minimal guidance on diagnosis of “child onset” disorders in adults (e.g., ADHD) and diagnosis of “adult onset” disorders in children (e.g., bipolar disorder). Disorders of infants and very young children are not covered well in ICD-10 and DSM-IV. This has led to the development of a separate diagnostic system called “Diagnostic classification of mental health and developmental disorders of infancy and early childhood”, better known as DC: 0-3 in the US More importance needs to be given for phenomenology of psychiatric disorders in the elderly.[9,14]

### Gaps in classification

These include areas like personality disorders, relational disorders, prodrome, high-risk conditions, etc. Problems with exclusion rules also need to be tackled (e.g., diagnosis of hyperkinetic disorder in pervasive developmental disorders in ICD-10).[9,14]

### “Death of phenomenology”

According to Andreason,[35] since the publication of DSM-III in 1980, there has been a steady decline in the teaching of careful clinical evaluation that is targeted to the individual person's problems and social context and that is enriched by a good general knowledge of psychopathology. She remarks that students are taught to memorize DSM rather than to elicit other potentially important or interesting signs and symptoms that are not included in DSM but are important to understand the patient as a whole. She terms it as “death of phenomenology”-an unintended consequence of operational diagnostic criteria.

### Difficulty of use in diverse populations and settings

Chinese classification of mental disorders-2R, which is largely based on ICD-10, excludes almost all the somatoform disorders, so that particular prominence can be given to the category of neurasthenia, which is one of the most frequent diagnoses in Chinese psychiatry.[8]

## REIFICATION FALLACY

DSM-IV acknowledges that “there is no assumption that each category of mental disorder is a completely discrete entity with absolute boundaries dividing it from other mental disorders or from no mental disorder.” But, the mere fact that a diagnostic concept is listed in an official nomenclature and provided with a precise operational definition tends to encourage us to assume that it is a “quasi-disease entity” that can be invoked to explain the patient's symptoms and whose validity need not be questioned.[16]

## PSYCHIATRY IS IN A STAGE OF FLUX

Dr. Allen Frances, who was chairperson of task force for DSM-IV, opined that we are at the epicycle stage of psychiatry where astronomy was before Copernicus and biology before Darwin. He expects that our “inelegant and complex current descriptive system will be replaced by simpler and more elegant models.”[36]

The core of our current dilemma is that we will never be certain where to draw the boundary between one syndrome and the next until we understand the underlying mechanisms, and we will have difficulty identifying these mechanisms unless we have the clinical boundaries in the right places first.[3]

## CHANGES IN DIAGNOSTIC CRITERIA: IMPLICATIONS FOR NEWER CLASSIFICATION SYSTEMS

Subsequent revisions of the ICD and DSM have been accompanied by an increase in the number of diagnoses. Since the publication of DSM-I, the number of identified diagnoses has increased by more than 300% (from 106 in DSM-I to 365 in DSM-IV-TR).[1]

Zimmerman and Spitzer remarked that a careful analysis of the increase in diagnostic labels suggests that it almost entirely represents greater specification of the forms of pathology, thereby allowing more homogeneous groups to be identified. But, the increased number of diagnoses has been criticized as broadening the concept of mental disorder leading to “medicalization” of normal human behavioral, cognitive or emotional patterns that previously would not have been identified as pathological.[1]

There are several groups that lobby for changes in diagnosis by clubbing or splitting existing diagnosis or creating new entities for various reasons. Although such concepts may seem exciting and path breaking, Jablensky and Kendell warn that all changes in definition have several disadvantages.[2]

- They are confusing to clinicians.
- Relevance of all previous clinical and epidemiological research to the disorder as it is now defined is uncertain.
- Involves tedious and costly changes in the content and detailed wording of diagnostic interviews and algorithms.
- Risks discrediting the whole process of psychiatric classification.

They feel that there ought to be a prejudice against minor changes as it will be as substantial as major changes. Many difficult decisions about the balance of advantage and disadvantages will therefore be required as we progress forward.[2]

## KENDELL'S CRITERIA FOR ASSESSING IMPROVEMENT IN TAXONOMY

Kendell[3] noted that there are five ways in which a new taxonomy may be regarded as an improvement.

1. Be more comprehensive
2. Be easier to use
3. Deal better with the issue of “clinical significance”
4. Have higher reliability
5. Have higher validity

## IMPACT OF NEUROSCIENCE AND GENETIC RESEARCH ON PSYCHIATRIC CLASSIFICATION

Molecular genetics and neuroscience will play an increasing role in the understanding of etiology and pathogenesis of psychiatric disorders. But, their impact on psychiatric classification will depend on social, cultural and economic forces that shape the public perception of mental illness and determine the practice of psychiatry. Diagnosis and treatment of prodrome and high-risk conditions (e.g., inheriting a susceptibility gene, strong family history, etc.) will involve complex ethical decisions. They also have the potential for misuse of psychiatric diagnosis for political purposes, eugenics and euthenics.

While acknowledging the possibility of advent of an eliminativist “mindless” psychiatry, which will be driven by biological models and Jettison psychopathology, Jablensky and Kendell felt that clinical psychiatry was likely to retain psychopathology. They further opined that a classificatory system with two major axes: One etiological, using neurobiological and genetic organizing concepts, and another syndromal or behavioural-dimensional was also possible.[2]

## IS THE FUTURE ICD OR DSM?

Radical changes are much more likely to be introduced by the APA than by the WHO, mainly because the former only has to persuade its own board of trustees, whereas the latter has to persuade the representatives of over 200 different countries at a formal revision conference.[2] Despite these problems, it is likely that both WHO and APA will try to reduce the number of minor differences between their respective classifications in future revisions.[2]

ICD-8 and DSM-II had a lot of similarities between them. Differences emerged with the publication of DSM-III. However, with joint initiatives, ICD-10 and DSM-IV have been brought together for several diagnoses. But, do we really need two classificatory systems that are trying to be compatible with each other instead of clubbing them into one? It is confusing to have two rival classifications, particularly because many of the differences between them are trivial, and in some cases, accidental.[2] But, the existence of two parallel nomenclatures and sets of definitions does help to emphasize that most of psychiatry's illness concepts are still provisional and that their definitions are arbitrary.[2]

It is jokingly said that DSM is the most widely read book after the bible. DSM is a blockbuster publication of the American psychiatric publishing and APA makes huge profits out of DSM sales. In contrast, WHO incurs major expenses for holding international conferences to arrive at a consensus for ICD. Besides, DSM gives APA considerable clout in world psychiatry as it is widely used in research and reputed journals require studies to use DSM classification for publication. With such enviable power that DSM gives APA, it is unlikely that APA will agree to club DSM with ICD. Thus, at least for the near future, both classifications are likely to continue to produce new editions or revisions and, in some respects, compete with one another.[2]

## CONCLUSION

Classificatory concepts are like hinges on which almost all empirical investigations turn. In psychiatry, as elsewhere, there are issues at a more fundamental level upon which the success of a classification depends. In short, a classification can only be as good as its underlying theory.[37]

Jaspers was prophetic when he remarked, “When we design a diagnostic schema, we can only do so if we forego something at the outset……and in the face of facts we have to draw the line where none exists…. A classification therefore has only provisional value. It is a fiction which will discharge its function if it proves to be the most apt for the time”.[38] His words hold true even today and will continue to do so for all subsequent classifications in the future.

We have made significant progress so far in understanding the disorders of the human mind and the future holds great promise in unraveling the mysteries further. Our classificatory systems will continue to evolve with advances in science. After all, change is the only thing permanent about this world and psychiatry classification is no exception.
